# Isolation and Culture of Human Stem Cells from Apical Papilla under Low Oxygen Concentration Highlight Original Properties

**DOI:** 10.3390/cells8121485

**Published:** 2019-11-21

**Authors:** Murielle Rémy, Francesca Ferraro, Pierre Le Salver, Sylvie Rey, Elisabeth Genot, Mojgan Djavaheri-Mergny, Noélie Thébaud, Claudine Boiziau, Hélène Boeuf

**Affiliations:** 1 INSERM, University Bordeaux, U1026, Laboratory for the Bioengineering of Tissues, F-33000 Bordeaux, France; murielle.remy@inserm.fr (M.R.); francesca.ferraro.bio@gmail.com (F.F.); pierre.le.salver@gmail.com (P.L.S.); sylvie.rey@inserm.fr (S.R.); noelie.thebaud@inserm.fr (N.T.); claudine.boiziau@inserm.fr (C.B.); 2 INSERM, University Bordeaux, U1045, F-33000 Bordeaux, France; elisabeth.genot@u-bordeaux.fr; 3 INSERM, University Bordeaux, U1218 Action, F-33000 Bordeaux, France; mojgan.mergny@inserm.fr

**Keywords:** SCAPs, low oxygen concentration, proliferation, differentiation, autophagy, CD49f, SSEA4, BNIP3

## Abstract

Stem cells isolated from the apical papilla of wisdom teeth (SCAPs) are an attractive model for tissue repair due to their availability, high proliferation rate and potential to differentiate in vitro towards mesodermal and neurogenic lineages. Adult stem cells, such as SCAPs, develop in stem cell niches in which the oxygen concentration [O_2_] is low (3–8% compared with 21% of ambient air). In this work, we evaluate the impact of low [O_2_] on the physiology of SCAPs isolated and processed in parallel at 21% or 3% O_2_ without any hyperoxic shock in ambient air during the experiment performed at 3% O_2_. We demonstrate that SCAPs display a higher proliferation capacity at 3% O_2_ than in ambient air with elevated expression levels of two cell surface antigens: the alpha-6 integrin subunit (CD49f) and the embryonic stem cell marker (SSEA4). We show that the mesodermal differentiation potential of SCAPs is conserved at early passage in both [O_2_], but is partly lost at late passage and low [O_2_], conditions in which SCAPs proliferate efficiently without any sign of apoptosis. Unexpectedly, we show that autophagic flux is active in SCAPs irrespective of [O_2_] and that this process remains high in cells even after prolonged exposure to 3% O_2_.

## 1. Introduction

Different stem cell models are under investigation for cell therapy purposes, all having their advantages and concerns. Among the different models, embryonic stem cells (ESCs), induced pluripotent stem cells (iPSCs) or adult stem cells, mesenchymal stem cells (MSCs), show great promise in the field of regenerative medicine [1]. Indeed, the clinical use of such cells is safe and without ethical restriction. MSCs display multipotent differentiation capacities and can be isolated from many sources (bone marrow, adipose tissue, skeletal muscle, hair follicle) including those of dental origin [2,3]. They are plastic-adherent cells, expressing CD (Cluster of Differentiation) 73, CD90 and CD105, but not CD14, CD34, CD45 or HLA-DR (Human Leucocyte Antigen-DR isotype) cell surface markers. MSCs are stored in many tissues as reparative cells, that can be mobilized and differentiated in response to trauma, disease, or aging [4].

A unique population of MSCs referred as SCAPs (stem cells from apical papilla), resides in the apical papilla of human immature wisdom teeth. SCAPs appear to be the source of odontoblasts that are responsible for the formation of root dentin [5,6]. Compared with dental pulp stem cells (DPSCs), they have a higher expression of telomerase, a superior proliferation rate and dental tissue regeneration capacity [6,7,8]. They express surface markers common to other dental stem cells, like STRO-1, but also a distinctive marker, CD24 [9]. SCAPs also express Survivin, a protein which plays a significant role in cell proliferation [7,10].

Like many adult stem cells, SCAPs originate from stem cell niches with a low oxygen concentration (<5% versus 21% in the ambient air). This parameter should be taken into consideration in order to exploit the full potential of stem cells [11,12,13]. Previous studies reported that DPSCs cultured for 24 h at 1% O_2_ secrete FGF2 (Fibroblast Growth Factor 2) and display improved survival rates when injected into experimental animals [14]. Recently candidate coding and non-coding RNAs, induced upon hypoxia, were reported in the dental pulp stem cell (DPSC) model [15]. Furthermore, plating bone marrow (BM) extracts directly at 3% O_2_ leads to the establishment of BM-MSCs, termed MIAMI cells, which have increased self-renewal and improved maintenance of osteoblastic differentiation capability [16,17,18]. In vitro studies revealed that BM-MSCs cultured under low oxygen concentration present high colony-forming potential [19], increased proliferation capacity [20] and better maintain their characteristics of undifferentiated cells. In addition, BM-MSCs cultured under low oxygen tension before their implantation, exhibit improved repair capabilities for bone defects, tendon tear, stroke and hindlimb ischemia [21,22]. Adipose tissue derived stem cells (ADSCs) proliferate well and maintain stemness with improved differentiation potential when grown under low O_2_ concentration [23,24].

Despite these studies, the role of oxygen concentration on MSC properties and therapeutic potential remain unclear, owing to differences in cell isolation methods, culture conditions and experimental strategies [25].

One well-known process regulated by low oxygen concentration ([O_2_]) is autophagy. Indeed, autophagy plays a key role in stem cell homeostasis as it acts as a robust quality control mechanism [26]. Autophagy is a highly conserved cellular process by which cytoplasmic components, including proteins, lipids and whole organelles, are sequestered into double-membrane vesicles called autophagosomes, then degraded and recycled upon the fusion of the autophagosome with lysosomes. Autophagy maintains cellular homeostasis under cellular stress conditions, such as endoplasmic reticulum stress, reactive oxygen species (ROS) accumulation, hypoxia or intracellular pathogen infection. It is usually activated in order to eliminate misfolded or aggregated proteins, to clear damaged organelles and to eradicate intracellular pathogens [26,27,28]. Dysfunctions in autophagy processes are associated with severe diseases, such as cancer and neurodegenerative diseases [29]. Autophagy-related (ATG) proteins such as microtubule-associated protein light chain 3 (LC3) are required for autophagosome formation. When autophagy is induced, cytosolic LC3 (LC3-I) is conjugated to phosphatidylethanolamine (PE) to form lipidated LC3 (LC3-II) which binds the autophagosomal membrane. As such LC3-II is a reliable marker of autophagy [30]. Specific drugs can be used to interfere with autophagic flux such as chloroquine, an anti-malarial drug which blocks autophagosome fusion with lysosomes, allowing amplification of the detection of cytoplasmic punctate staining of LC3 by immunofluorescence. Bcl2 (B cell leukemia/lymphoma 2)/adenovirus E1B 19 kDa protein-interacting protein 3 (BNIP3), a HIF1α (Hypoxia Inducible Factor 1α) target that is induced upon hypoxia, is a mitophagy regulator which can be associated with LC3-II on the autophagosome during mitophagy. BNIP3 has been also shown to regulate apoptosis [31,32,33].

Human immature wisdom teeth are commonly extracted in teenagers for orthodontic reasons. In this study, we compared the properties of SCAPs (isolated from three teenagers) that were isolated and expanded in parallel under continuous low or high [O_2_] conditions. We also assessed the impact of long-term growth (up to one month) at low [O_2_] on these properties. This study established the advantage and limitations of SCAP isolation and expansion at low [O_2_].

## 2. Materials and Methods

The study was conducted in accordance with the Declaration of Helsinki and after approval of the French Research Ministry (DC 2008-412). Wisdom teeth were collected at the Unité de Médecine Bucco-Dentaire—Groupe Hospitalier Saint-André—Centre Hospitalier Universitaire (CHU) of Bordeaux (France), according to the procedure approved by French regulations. All teeth were collected with informed and oral consent from the patients according to the ethical guidelines set by the French law.

### 2.1. Tooth Recovery

Wisdom teeth were collected from healthy teenagers (male, 15-year-old) because of orthodontic indications. Since the quality of teeth obtained could depend on various parameters linked with the “way of life” we applied some exclusion parameters as follows: teeth were excluded from individuals who drank more than two glasses of wine, beer or one glass of strong alcohol twice a week, smoked more than six cigarettes/day and took any illegal drugs. Teeth were chosen because of their root immaturity taking into account the age of the patient and panoramic radiography, an essential complementary exam for wisdom tooth extraction. Before extraction, the mouth was cleaned with Povidone iodine solution. After the classic procedure used for germectomy, the decontamination was done again, teeth with immature roots were removed out of their alveolus and immediately immersed in pre-warmed and pre-equilibrated 3% or 21% O_2_ transportation medium (α-MEM (Minimum Essential Medium), Gibco A10490-01) containing 10 mg/mL antibiotics (penicillin, streptomycin (Gibco 15140-122)), directly in the surgical room. Immature teeth were never treated with NaOCl (above 1.5%), shown to be detrimental for SCAP survival [34] and were exposed only a few seconds to ambient air. Only patients with more than one wisdom tooth to be extracted were chosen in order to allow the establishment of SCAPs in parallel at 21% O_2_ and 3% O_2_.

### 2.2. Experimental Settings

Experiment I (EXP I, for preliminary results): SCAPs from the tooth of one teenager were isolated under 21% O_2_, grown for 10 days in α-MEM supplemented with 10% fetal bovine serum (FBS, Biowest, Les Ulis, France—LOT N° S1390351810) with one passage (cells were diluted at 1/3) and frozen in cold freezing medium containing 90% FBS/10% DMSO (1 million cells/mL). These batches were designated as “passage 1” (P1). Cells were then thawed under atmospheric air conditions and passaged into two T25 flasks: one incubated at 21% O_2_ and the other incubated at 3% O_2_. Cells were then subsequently passaged twice a week, counted and phenotyped by flow cytometry analysis using classical MSC markers.

Experiment II (EXP II, for preliminary results): SCAPs from the tooth of one teenager were isolated under 3% O_2_, split into two T25 flasks: one incubated at 21% O_2_ and the other one at 3% O_2_. Cells were then passaged twice a week, counted and phenotyped by flow cytometry analysis using classical MSC markers. This experiment was reproduced with three individuals.

**Figure 1:**
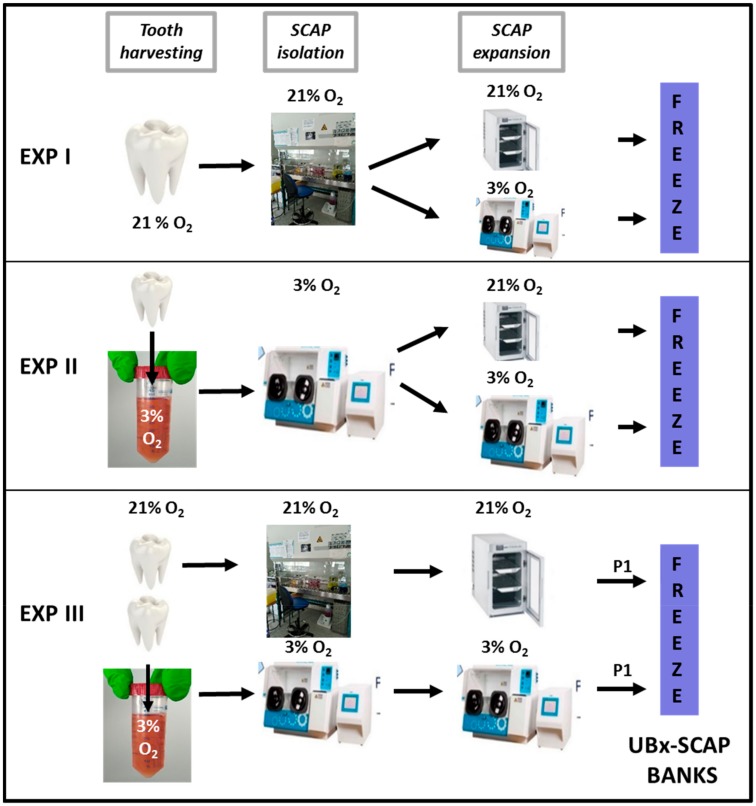
Design of the experiments. Diagram of the different experimental procedures (EXP I, II, III) is shown with the percentage of O_2_ for tooth harvesting, stem cells from apical papilla (SCAP) isolation and expansion. In Experiment (EXP) III, two teeth from the same individual were harvested and processed at 21% or 3% O_2_. SCAPs derived from EXP III were named: UBx-SCAP. SCAPs at 21% O_2_ were named: UBx-SCAP-N1, N2 and N3. SCAPs at 3% O_2_ were named: UBx-SCAP-H1, H2 and H3.

Experiment III (EXP III): SCAPs from two teeth from the same patient were isolated and expanded at either 3% or 21% O_2_. At the end of the amplification step (including one passage), cells were frozen. After thawing, cells were grown and experiments performed at the same [O_2_] as that used for their isolation. SCAPs were isolated from three different individuals using these conditions and named thereafter: UBx-SCAP-N1, N2 and N3 (21% O_2_) or UBx-SCAP-H1, H2 and H3 (3% O_2_). These settings are summarized in Figure 1. The controlled [O_2_] cell chamber was an Invivo2 300 RUSKINN in which all experiments at low O_2_ concentration were performed. When stipulated, early passages were up to P7 and late passages were from P11 up to P22. The detailed experimental procedure for the preparation of SCAPs for EXP III is provided below.

### 2.3. Detailed UBx-SCAP Preparation

Freshly extracted teeth were directly put in transportation medium and brought to the laboratory in a pre-warmed pocket at 37 °C. For cells derived under 3% O_2_, the medium (40 mL) was conditioned for 1 h under 3% O_2_. Teeth were then processed in a sterile hood at 21% or 3% O_2_ as previously described [6,35]. Briefly, root apical papilla were gently separated from the surface of the root using a dental forceps and maintained in HFF solution (Hank’s Balanced Salt Solution (HBSS, Gibco—14025-050, Thermo Fisher Scientific, Illkirch, France) supplemented with 5% of Fetal Bovine Serum). Apical papillae were minced in small pieces, rinsed in HFF solution and pelleted by centrifugation (2000 rpm for 5 min). Cell pellets were resuspended and incubated in a 10 mL solution of 1:1 collagenase (Sigma Aldrich–C26741G, Merck, Saint Quentin Fallavier, France, 125 CDU/mg) (6 mg/mL) and dispase (Gibco 17105-041 1.88 units/mg) (8 mg/mL) at 37 °C, for 1.5 h and under agitation. The digested mixture was washed with 40 mL of HFF solution, centrifugated, resuspended in 20 mL of HFF and filtered with a 40 µm strainer. Cells were seeded into 25 cm^2^ culture flasks and cultured with 7 mL of α-MEM supplemented with 20% FBS, penicillin–streptomycin and gentamycin (5 mg/mL). The medium was refreshed on the following day and then twice a week. For the subsequent passages, cells were cultured and expanded in 75 cm^2^ culture flasks in the amplification medium (α-MEM supplemented with 10% FBS, penicillin–streptomycin or gentamycin (0.5 mg/mL)) and frozen at sub-confluency (P1). For the experiments, thawed cells were expanded in 75 cm^2^ flasks in the amplification medium and were passaged twice a week. For all experiments performed at 3% O_2_, the medium was preconditioned for 1 h at 37 °C, 5% CO_2_ and 3% O_2_.

### 2.4. Cell Proliferation Assay

SCAPs were plated in amplification medium in 75 cm^2^ culture flasks at a density of 5300 cells/cm² under 3% O_2_ (0.4 million cells per flask) and 10,600 cells/cm² (0.8 million cells per flask) under 21% O_2_, changed after 48 h and counted after a four-day culture period. These cell seeding conditions were chosen for practical reasons, to keep the cell passaging step on the same day, since cells cultured under 3% O_2_ were growing faster than those cultured at 21% O_2_. Two parameters were calculated to characterize the cell expansion:

Doubling population time (DPT) was obtained with the formula:

(1) $$\mathrm{DPT}=\frac{\log\left(2\right)\times \mathrm{number}\;\mathrm{of}\;\mathrm{hours}\;\mathrm{in}\;\mathrm{culture}}{\log\;\left(\mathrm{counted}\;\mathrm{cells}\right)-\log\;\left(\mathrm{seeded}\;\mathrm{cells}\right)}$$

Population doubling was determined by the equation [36,37]:

(2) $$\mathrm{PD}=\frac{\log\;\frac{\left(\mathrm{counted}\right)}{\left(\mathrm{seeded}\right)}\mathrm{cells}\;}{\log\;\left(2\right)}.$$

The cumulative population doubling is the total number of doublings from the start to the end of the culture period and was used to draw the proliferation curves.

### 2.5. Colony Forming Unit (CFU) Assay

Cells grown at 3% or 21% O_2_ were used at early passage (P2) and late passage (between P11 to P22). They were seeded at a clonal density of 5 cells/cm^2^ of a six-well plate in α-MEM supplemented with 10% fetal bovine serum (FBS) and incubated under a humidified atmosphere of 5% CO_2_ at 21% O_2_. After seven days, cells were fed with fresh medium for an additional period of seven days. At day 14, colony formation was obvious and the assay was stopped by fixation with cold absolute methanol for 10 min at 4 °C. Colonies were stained for 10 min in 0.5% crystal violet prepared in 25% methanol. After washing, colonies with more than 50 cells were scored by the use of a Leica MZ10F magnifying device. Results were analyzed by calculation of cloning efficiency E (E (%) = (Number of colonies/number of seeded cells) × 100).

### 2.6. Cell Cycle Analysis

Cells were trypsinized, washed once with PBS, counted and 1 million cells was resuspended in 100 µL of PBS buffer. Cold ethanol (900 µL, 70% in water) was added dropwise to the cell suspension and left for 1 h at room temperature. After two washes with 10 mL of PBS, the pellet was homogenized in 250 µL of PBS containing RNase A (Sigma-Aldrich, Merck, Saint Quentin Fallavier, France, R-6513, final concentration 0.5 mg/mL) and incubated for 1 h at 37 °C. Propidium iodide solution (Sigma-Aldrich P-4170) was added at 10 µg/mL final and the suspension was kept in the dark until flow cytometry using a 488 nm laser.

### 2.7. Flow Cytometry Analysis of Membrane Markers

SCAPs at early passage, from P2 to P7 (*n* ≥ 6) were analyzed by flow cytometry for expression of specific membrane markers. Antibodies were fluorochrome-coupled antibodies (Table 1).

SCAPs were trypsinized, re-suspended in complete medium, counted and fixed (1 million cells/mL in PBS containing 1% PFA) for 15 min at 4 °C. After two washes with 10 mL PBS, samples were incubated with PBS containing 1% bovine serum albumin (BSA, blocking solution) for 30 min at room temperature. Cells were then resuspended in the blocking solution and dispatched at 1 million cells per test condition. The assay was performed by incubating 1 μL of each antibody in 100 µL of cell suspension in the blocking solution for 1 h at room temperature, washed twice in PBS, re-suspended in 200 μL of PBS and kept on ice until flow cytometry analysis. For each experiment, isotype controls coupled with the same fluorochromes (Table 1) were included to calibrate the cytometer. Analyses of the samples were performed with a Bioscience BD Accuri C6 Flow Cytometer. Data were analyzed using CFlow Plus software: for each passage and oxygen condition 10,000 events were gated. The percentage of positive cells and the mean fluorescence intensity (MFI, in arbitrary units) were recovered and analyzed.

### 2.8. Differentiation Procedures

For osteogenic and adipogenic differentiation, cells were plated at a density of 5000 cells/cm^2^ in 2 mL of control medium (MEM containing 10% FBS) and differentiation was started the day after plating with the differentiation cell kit media: StemPro^®^ Osteocyte/Chondrocyte Differentiation Basal Medium (Gibco—A10069-01) containing StemPro^®^ Osteogenesis Supplement (Gibco—A10066-01) or StemPro^®^ Adipogenesis Differentiation Basal Medium (Gibco—A10410-01) containing StemPro^®^ Adipogenesis Supplement (Gibco—A10065-01).

For chondrogenic differentiation a drop containing 80,000 cells was seeded in the well and after 2 h the control or chondrogenic media were added: StemPro^®^ Osteocyte/Chondrocyte Differentiation Basal Medium (Gibco—A10069-01) containing StemPro^®^ Chondrogenesis Supplement (Gibco—A10064-01).

Osteogenic differentiation was revealed after three weeks with OsteoImage™ kit (PA-1503—Lonza, Basel, Switzerland). OsteoImage staining reagent labelled the hydroxyapatite portion of the bone-like nodules formed by osteoblasts. Pictures were taken with the confocal microscope Leica TCS SPE equipped with the Leica CTR6500 Electronic Box. Chondrogenic differentiation was assessed after three weeks with Alcian Blue staining and adipogenic differentiation with red oil staining. Positive controls for each differentiation processes were performed with human bone marrow mesenchymal stem cells (hBM-MSCs) and adipose tissue derived stem cells (ADSCs, from a human liposuction) (data not shown).

For all the experiments performed with SCAPs, the differentiation processes were performed under 21% or 3% O_2_, as for cell isolation.

### 2.9. Western Blotting and Immunolabelling

Primary antibodies for Western blots and immunolabeling are listed in Table 1.

For Western blotting: Nanog (1:500), Oct4 (1:500), ERK2 (Extracellular Signal-Regulated Kinase 2) (1:1000). Cell lysates were prepared in RIPA buffer supplemented with protease inhibitor cocktail (Sigma-Aldrich, P8340) and Halt phosphatase inhibitor cocktail (Thermo Fisher Scientific, Illkirch, France), (0.5 mL of buffer per T25 flask containing sub confluent cells, after four days in culture), scraped and centrifugated for 20 min at 10,000 rpm. Protein concentrations of the clarified lysates were established by the Pierce BCA protein assay kit (Thermo Scientific, 23227). Protein lysates (50 µg) were loaded onto a 10% acrylamide/bis protein gel, transferred onto nitrocellulose membrane with the Turbo transfer apparatus (Biorad, Marne-la-Coquette, France) and hybridized with the antibodies as previously described [38,39]. ERK2 was used as a loading control.

For immunocytochemistry: BNIP3 (1:400) and LC3B (1:150). Secondary antibodies (diluted 1:1000) were either AlexaFluor 488 or AlexaFluor 568, Table 1. 10,000 cells (under 3% O_2_) and 20,000 cells (under 21% O_2_) were seeded in 24 multi-well plates (high resolution plates, IBIDI, Ibitreat, 82406), grown for four days, treated or not with 20 µM chloroquine (Sigma-Aldrich, C6628) for 5 h and fixed with 4% PFA in PBS for 15 min at room temperature. Cells were first labelled with the anti-LC3 antibody: after two washes in PBS, cells were washed with PBS–0.1% gelatin (Sigma Life Science, G1890) and permeabilized with 50 µg/mL digitonin (Sigma-Aldrich, D6628) in PBS–0.1% gelatin for 10 min at room temperature. Then cells were washed in PBS–0.1% gelatin for 5 min on a rocker and then incubated with PBS–0.1% gelatin for 30 min at room temperature. Cells were incubated with the primary anti-LC3 antibody (dilution 1:150 in PBS–0.1% gelatin) overnight at 4 °C. After one wash in PBS–0.1% gelatin, for 5 min, cells were incubated with the secondary antibody (goat anti-mouse-coupled to Alexa 488 (diluted 1:1000) in PBS–0.1% gelatin), for 1 h at room temperature. Then cells were washed in PBS–0.1% gelatin for 5 min and then two times in PBS for 5 min. Cells were counterstained with DAPI (4′,6-Diamidine-2′-phenylindole dihydrochloride, Thermo Scientific-SG2423831, 1 µg/mL). Pictures were taken with the confocal microscope Leica TCS SPE with the Leica CTR6500 Electronic Box. Cells were then stained with an anti-BNIP3 antibody with an optimized procedure [40]. Briefly, cells were permeabilized in 0.3% Triton-X100 in PBS for 15 min at room temperature, washed once with PBS and incubated with the blocking solution (PBS containing 0.1 % of BSA, 10% of goat serum, 0.2% Triton X-100 and 0.05% Tween-20) for 1 h at room temperature. Anti-BNIP3 antibody diluted in the blocking solution was incubated overnight at 37 °C. After two washes with PBS, cells were incubated with the secondary antibody, AlexaFluor 568 anti-rabbit diluted 1:1000 in the blocking solution for 2 h at 37 °C. Cells were washed with PBS and imaged with the same confocal microscope.

### 2.10. Statistical Analyses

Data are presented as mean ±standard deviation (SD). Differences between groups were evaluated using Mann Whitney for two compared groups for non-parametric measures. Two-tailed *p* values less than 0.05 were considered significant.

## 3. Results

### 3.1. SCAPs Display a Proliferative Advantage When Grown at 3% O_2_ Versus 21% O_2_

To test the impact of O_2_ concentration on SCAP properties, we set up different procedures for their isolation referred to EXP I, II and III (Figure 1). In EXP I, SCAPs isolated at 21% O_2_ were plated in two flasks incubated either at 21% O_2_ or 3% O_2_, after thawing at 21% O_2_. Routine microscopic observation and cell counting indicated that cells cultured under low [O_2_] grew faster (about 1.5-fold) than under 21% O_2_. We also noticed that SCAPs isolated directly under low [O_2_], (EXP II), grew faster: doubling population times were 50 h at 21% O_2_ and 31 h at 3% O_2_ and cumulative population doubling were higher at 3% versus 21% O_2_ (as shown in Figure S1). However, since the isolation procedures (EXP I and EXP II, Figure 1), were performed with teeth from distinct individuals, it remained possible that the differences observed between EXP I and II were not only O_2_-dependent but also individual-dependent.

Therefore, to determine whether it was the isolation process (at 21% or 3% O_2_) or only the expansion process (at 21% or 3% O_2_) which was important to improve proliferative efficacy, we undertook EXP III with SCAPs isolated from the same individuals, isolated and grown in parallel under 3% and 21% O_2_ (Figure 1).

**Figure 2:**
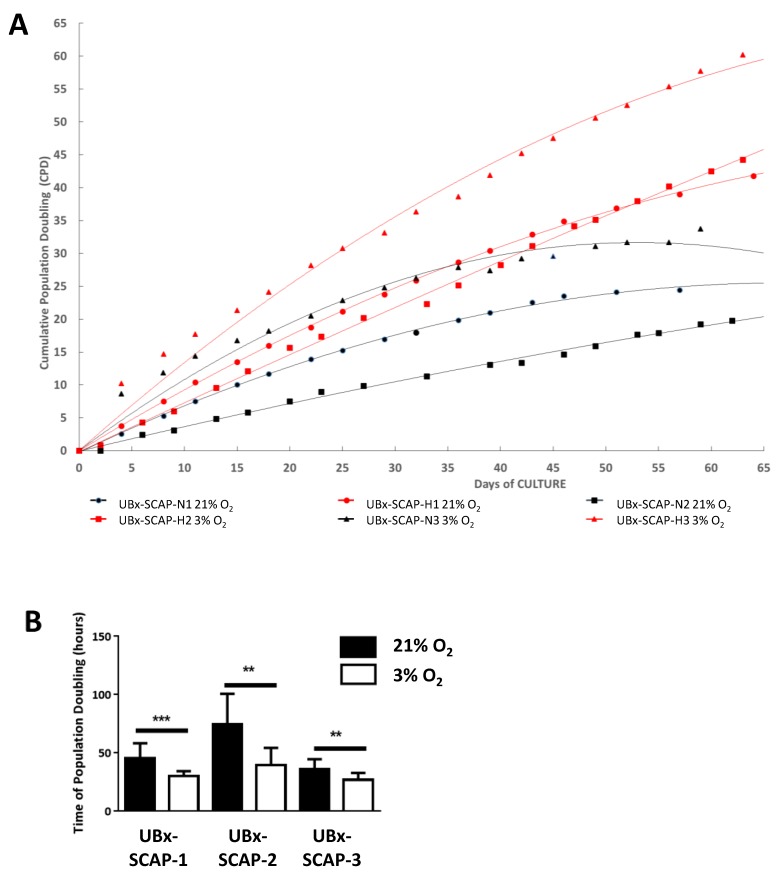
Proliferative advantage of UBx-SCAP isolated under 3% O_2_ in comparison with ambient air (21% O_2_). (**A**) At each passage of SCAPs from EXP III, 0.4 (under 3% O_2_) or 0.8 (under 21% O_2_) millions of cells were seeded in a 75 cm^2^ flask and counted after three or four days. Cumulative population doublings (CPD) were plotted for each individual refered to UBx-SCAP-N1, N2 and N3 (21% O_2_) and UBx-SCAP-H1, H2 and H3 (3% O_2_), up to 65 days. (**B**) The mean of time of population doubling for the first 10 passages, for each individual at 21% and 3% O_2_ is plotted with standard deviation. Statistical analyses were done with a Mann-Whitney test. ** *p* < 0.01. *** *p* < 0.001.

For the three individuals, we observed a higher proliferation rate when SCAPs were isolated and cultured at 3% O_2_ versus 21% O_2_ (Figure 2A). Significant differences in the time of population doubling were clearly observed, indicating an advantage to isolate SCAPs under 3% O_2_ (Figure 2B). Obviously, there were variations in the kinetic curves between the three individuals, linked to their genetic differences. However, the proliferative advantage at 3% O_2_ was clearly observed for each SCAP preparation. To determine whether the proliferative advantage could be linked to an increase in the proportion of cells in the S phase of the cell cycle, as documented in embryonic stem cells [41], we performed cell cycle analysis. The proportion of cells in S phase was slightly increased at low [O_2_] at early passage of EXP II and III, but the difference was too low and therefore unlikely to account for the increase in proliferation rate of cells at 3% O_2_ (Figure S2).

### 3.2. Clonogenicity of SCAPs In Vitro

**Figure 3:**
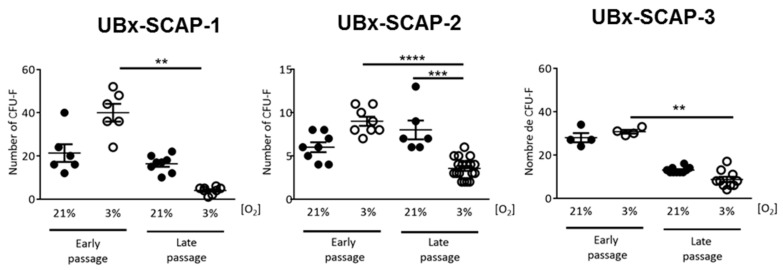
Clonogenicity tests. Plots of the number of colony forming units obtained after two weeks of culture at 21% O_2_, for 50 UBx-SCAPs initially isolated and grown at either 21% or 3% O_2_ with a low (<7) or high (>10) number of passages, as indicated. Statistical analyses were done with a Mann Whitney test. ** *p* < 0.01. *** *p* < 0.001. **** *p* < 0.0001.

The clonogenicity efficiency of MSCs grown at low [O_2_] has been reported to be improved in comparison with 21% O_2_ [42,43]. We developed an assay to determine whether improved clonogenicity was maintained when cells were switched from 3% to 21% O_2_. Therefore, we analyzed the clonogenicity of early and late passages of SCAPs, isolated at 21% or 3% O_2_ and grown at 21% O_2_. The total number of clones obtained, after seeding 50 cells (as detailed in Materials and Methods), was plotted for each patient, at early and late passages (Figure 3). First, we noticed that the number of clones obtained was different amongst patients. Also, the clone number recovered at 3% O_2_, for UBx-SCAP-1 and 2 was slightly higher than at 21% O_2_, but not significantly so. At late passages there was a significant decrease in the number of clones obtained at 3% O_2_ versus 21% O_2_, for the three UBx-SCAPs. These data indicate that the switch from 3% O_2_ to 21% O_2_, as performed in this assay, did not alter clonogenicity at early passage but was rather deleterious for clonogenicity at late passage.

### 3.3. Differential Expression of Membrane Markers

**Figure 4:**
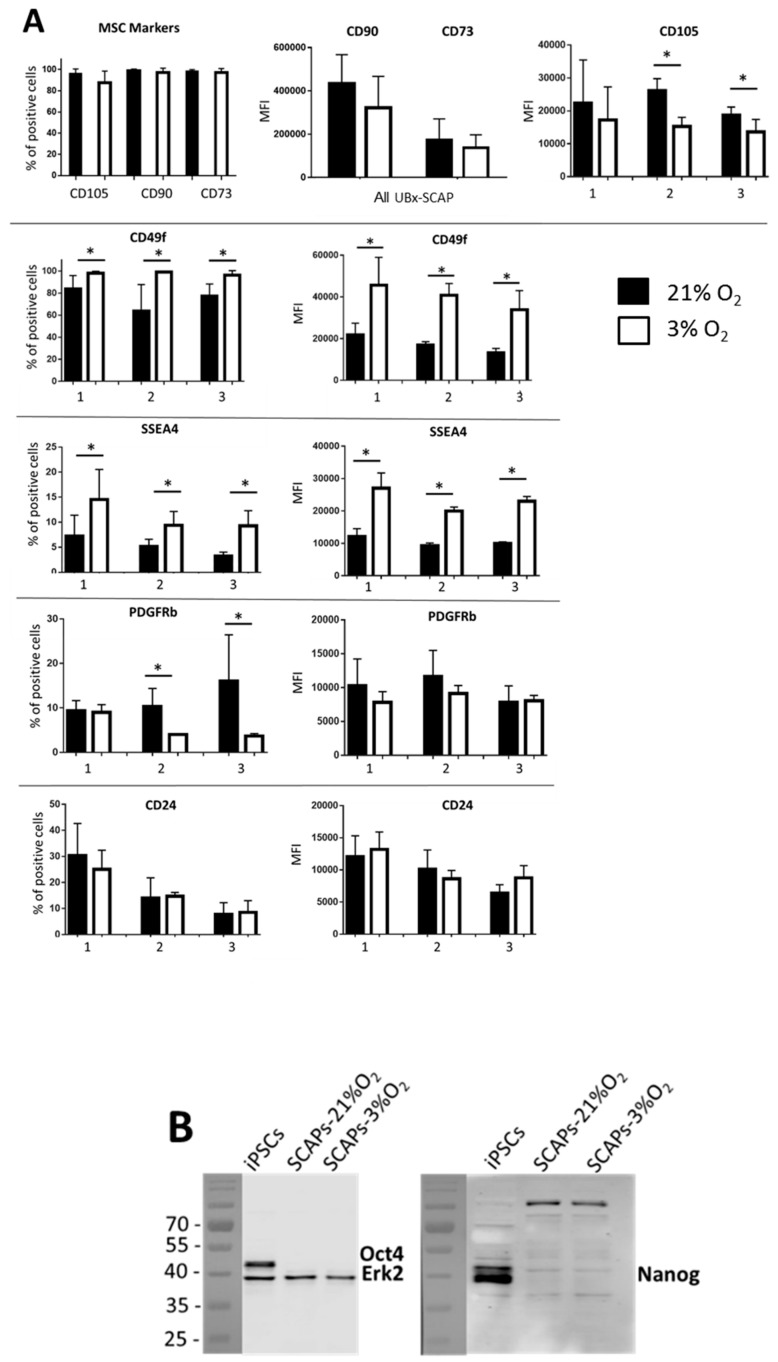
Protein expression analysis. SCAPs from EXP III (early passages of UBx-SCAP) were grown at 3% or 21% O_2_. (**A**) Graphs showing flow cytometry analysis of different markers as indicated ((% of positive cells, left column) and mean of fluorescence intensity (MFI, in arbitrary units, right column)). Numbers 1, 2, 3 refer to the three individuals. For each group, three to six samples from different early passages were analyzed. Statistical analyses were done with a Mann Whitney test. * *p* < 0.05. (**B**) Western blot analysis of UBx-SCAP-1 (early passage) grown under 21% or 3% O_2_ and of human iPSCs (induced pluripotent stem cells, IMR90 cell line) used as a positive control for expression of Oct4 and Nanog. ERK2 was used as the loading control.

We investigated the expression of classical MSC markers (CD73, CD90, CD105) and also that of other markers described as “stemness/proliferative-linked” markers like CD49f, stage-specific human embryonic antigen-4 (SSEA4), CD24 and PDGFRβ [44,45,46,47]. We observed that the MSC phenotype was not affected by the [O_2_] since the percentage of positive cells for the classical MSC markers was similar in both conditions (Figure 4A). However, we observed a slight decrease in the MFI of the CD105 marker at 3% O_2_ as previously reported [43], while the MFI of CD90 and CD73 remained unaffected by the [O_2_], independently of patient and cell passage. In contrast, both the percentage of positive cells and the MFI of CD49f and SSEA4 were consistently higher, for the three patients at 3% O_2_ compared to 21% O_2_ (Figure 4A). Expression of CD24 was rather patient- than O_2_-dependent and the percentage of PDGFRβ positive cells was slightly decreased under low [O_2_]. Irrespective of the condition tested, we did not detect any positive cells for CD45 and CD34, as expected, nor for CD271, that was previously reported to be present in a small population of MSCs [48] data not shown). In addition, we also analyzed, by Western blot, the expression of embryonic stem cell markers like Nanog and Oct4, shown to be expressed in ADSCs and MIAMI cells [18,24]. Under our culture conditions, these markers were not expressed in SCAPs irrespective of cell passage and [O_2_], while they were highly expressed, as expected, in human induced pluripotent stem cells (iPSCs), which were used as positive controls (Figure 4B) [49]. 

### 3.4. SCAPs Differentiate into the Three Mesodermal Lineages but Lose Potential at Late Passages under Low [O_2_]

**Figure 5:**
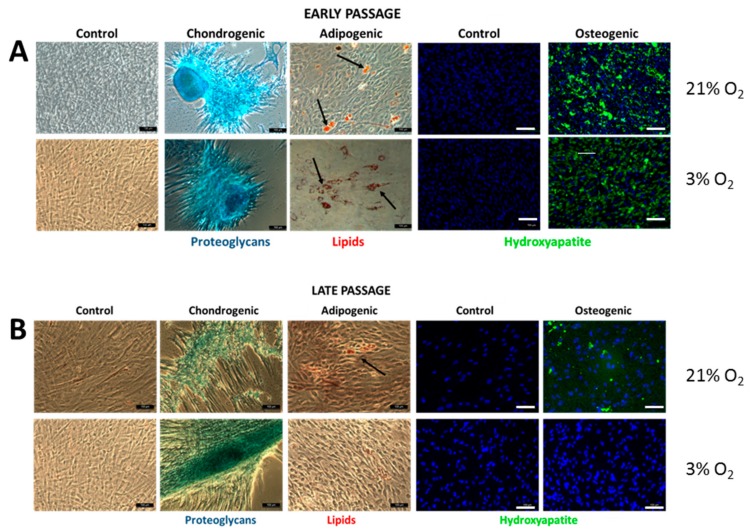
Differentiation of SCAPs. SCAPs from EXP III initially isolated and grown at 3% or 21% O_2_ (from early (**A**) or late passages (**B**)) were cultured at the same oxygen concentration in control or differentiation media. Representative pictures of experiment performed with UBx-SCAP-3 are shown: Alcian blue (chondrogenic differentiation), red oil (adipogenic differentiation, shown by arrows) and OsteoImage (osteogenic differentiation revealed by green fluorescent dots of HA—hydroxyapatite deposits) staining are shown. Cells from early and late passages are shown as indicated. Scale bar is 100 µm.

To determine whether SCAPs grown under 21% or 3% O_2_ behave as classical MSCs, we undertook in vitro differentiation experiments towards mesodermal lineages. Cells from the three individuals at early or late passages were induced to differentiate as described in the Materials and Methods, then stained with an OsteoImage™ kit (which reveals the formation of hydroxyapatite mineralized matrix by osteoblasts), with red oil (to reveal the presence of adipocytes) or Alcian blue (for staining of glycosaminoglycans produced by chondrocytes). As shown in Figure 5, cells at early passages could be differentiated into the three cell lineages and no effect of [O_2_] was detected, indicating that cells kept their stemness properties in both conditions in vitro. At later passages, we observed a loss in osteogenic and adipogenic differentiation potential at 3% O_2_, while chondrogenesis differentiation remained, indicating that low [O_2_] might preserve this unipotent mesodermal feature of SCAPs.

### 3.5. Autophagy is Increased under Low [O_2_] with Efficient Flux at Both 3% O_2_ and 21% O_2_

**Figure 6:**
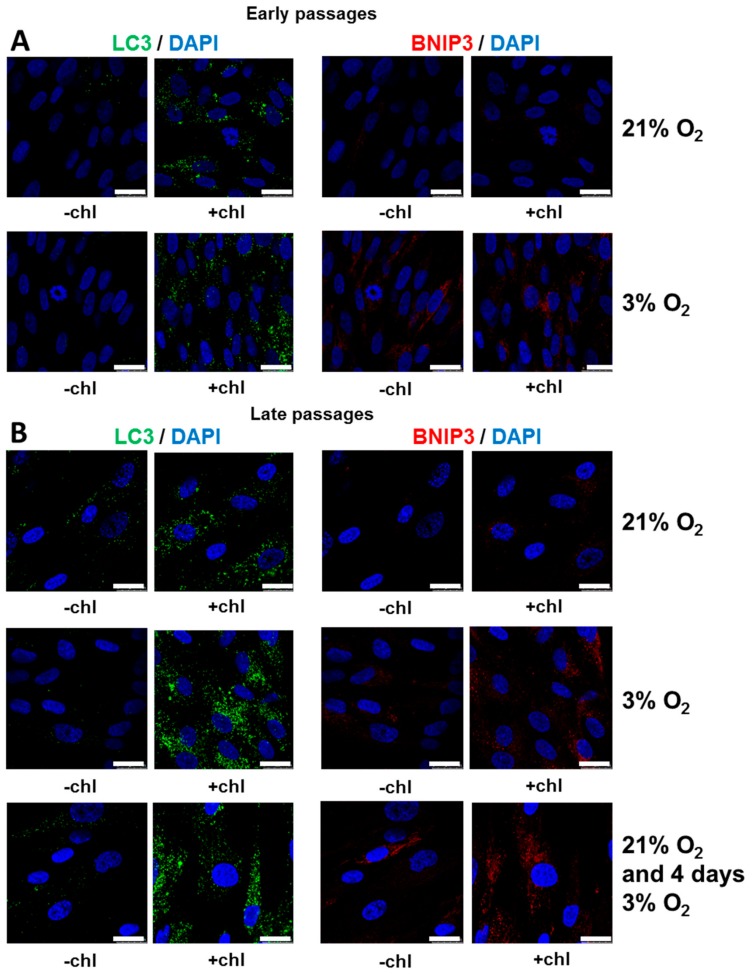
Characterization of the autophagy process. (**A**) Early and (**B**) late passages of SCAPs, isolated and expanded either at 21% O_2_ or at 3% O_2_, or isolated and expanded at 21% and switched for four days at 3% O_2_, were labelled with anti-LC3 or anti-BNIP3 antibodies as indicated. For each patient and condition cells were treated (+chl) or not (−chl) for 5 h with 20 µM chloroquine before labeling. Representative pictures of UBx-SCAP-3 are shown here. Careful analyses of DAPI stained nuclei did not reveal fragmented chromatin, a feature of apoptosis. Scale bar is 25 µm.

Recent studies have demonstrated that autophagy is involved in the maintenance of cellular stemness and in the differentiation of mesenchymal stem cells [50]. Therefore, it was interesting to determine the autophagy status of SCAPs as it has not been analyzed to date. Autophagosomes, the vesicles formed during the autophagy process, were detected by using a specific antibody against LC3, a marker of autophagy, as evidenced by punctate staining in the cell cytoplasm. Autophagic flux was monitored by comparing the amount of the LC3 puncta in the presence and absence of chloroquine, an inhibitor of lysosomes that prevents LC3 degradation, thus leading to the accumulation of LC3 puncta. As shown in Figure 6, the abundance of LC3 puncta was low at 21% O_2_ and 3% O_2_ without chloroquine but highly increased after the addition of chloroquine. These results demonstrated that the autophagic flux was well activated in both O_2_ conditions, at early (Figure 6A) as well as at late passage (Figure 6B) and for short (four days) as well as after long periods under 3% O_2_. However, at late passage there was an increase in LC3 punctate staining at 3% O_2_ (Figure 4B). We have also shown that the expression level of BNIP3, known to be involved in the mitophagy process [31,32], was highly increased at 3% versus 21% O_2_ (Figure 6A,B). Altogether, these results suggest that high autophagic flux was maintained at both O_2_ concentrations at both early and late passages, with mitophagy only being induced at 3% O_2_. Notably, we have also reproducibly observed heterogeneity in the LC3/BNIP3 staining, at both early and late passages by double staining of cells with these two antibodies. Indeed, as shown in Figure S3, cells could be LC3^+^/BNIP3^−^, LC3^−^/BNIP3^+^, LC3^+^/BNIP3^+^ and LC3^−^/BNIP3^−^ suggesting subtle different properties of SCAPs, linked with autophagy processes that will deserve further analysis.

## 4. Discussion

**Figure 7:**
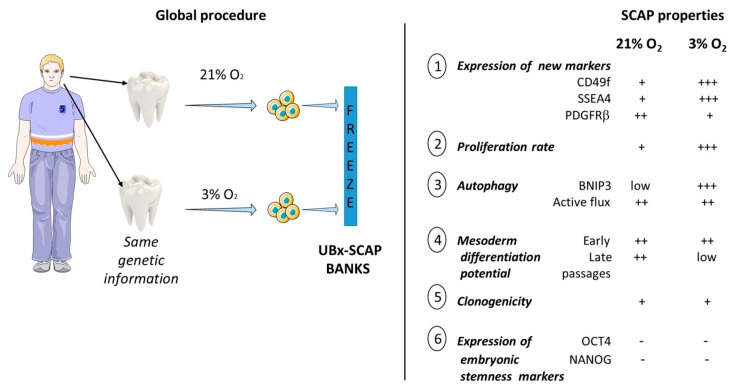
Summary of the salient features of UBx-SCAP harvested, isolated and expanded under 3% O_2_ compared with SCAP at 21% O_2_ (e.g., EXP III). We have developed a unique new model of adult teeth-derived stem cells (obtained from three individuals) and characterized their properties under different O_2_ concentrations, towards potential applications in regenerative medicine. SCAPs at 21% O_2_ were named: UBx-SCAP-N1, N2 and N3 and SCAPs at 3% O_2_ were named: UBx-SCAP-H1, H2 and H3.

Stem cells used for regenerative medicine should be well characterized in terms of their safety and optimal properties like high survival, expansion and differentiation potentials after transplantation [51]. Moreover, mimicking as best as possible their native microenvironment should promote optimal regenerative responses. The direct comparison of SCAPs harvested, isolated and amplified in parallel at ambient air versus low [O_2_] has not been reported previously nor has the impact of long-term culture (up to one month) at low [O_2_]. This study, performed with SCAPs from three individuals allowed a large spectrum of analyses regarding their properties such as proliferation, clonogenicity, cell cycle, multipotency and autophagic flux both at early and late passages (summarized in Figure 7).

Previous analyses performed with stem cells and in particular with BM-MSCs show many advantages for cells grown under low [O_2_] [21,52,53,54]. In addition, it is shown that MIAMI cells can promote better tissue repair and functional recovery in animal models, in comparison with cells grown at 21% O_2_ [18,55]. However, the process of collecting BM is invasive and we therefore reconsidered the use of more easily available MSCs with the aim of improving their expansion and repair capabilities. We focused on the importance of the [O_2_] parameter, starting with tissues which have never been exposed to ambient air, as depicted in this study. Wisdom teeth are often removed in teenagers for orthodontic reasons and could represent an adult stem cell source for future applications in regenerative medicine. Therefore, SCAPs were isolated from three teenagers and banked at 21% and 3% O_2_. This O_2_ concentration was chosen based on our initial experiments (EXP I and II) in which we observed high proliferation and no apoptosis of cells (based on visualization of intact DAPI stained nuclei) after at least one month at 3% O_2_.

### 4.1. High Proliferative Capacity but Not Better Clonogenicity under Low [O_2_]

We have shown that the cell isolation and expansion at low [O_2_] led to an increased proliferation capability of SCAPs and to a preserved expression of the classical MSC markers with a slight decrease of CD105, as already reported [43]. A recent report with DPSC isolated at 21% O_2_ and expanded at either 21% or 3% O_2_ (as in our EXP II) also demonstrated an advantage of cell growth under low [O_2_] compared with ambient air [15]. Among the different markers analyzed, CD49f and SSEA4 remained highly expressed under low [O_2_] in comparison with ambient air. In contrast, PDGFRβ, which is enriched in human placental MSCs with elevated colony forming unit efficiency [45], was moderately expressed in SCAPs irrespective of cell growth conditions and the percentage of positive cells was rather decreased under low [O_2_]. In addition, CD24 expression, which may refer to the number of committed progenitors cells in the papilla tissue, was not differentially expressed in our cell growth conditions [44]. Therefore, our study identified two new [O_2_]-dependent markers. CD49f/6 integrin, whose deletion in mouse models leads to a disabling skin disease (epidermolysis bullosa), is part of the laminin receptor in epithelial cells which plays a critical structural role in the hemidesmosome [56,57]. CD49f is associated with multipotency and maintenance of stemness in umbilical cord blood-derived MSCs through regulation of Oct4 and Sox2 [47]. It is also upregulated in skeletal stem cells (SSC) as compared to fibroblasts and this marker has been associated with functional SSCs [58]. However, CD49f-positive BM-MSCs showed higher clonogenicity and differentiation capacity in vitro than CD49f-negative BM-MSCs [59], a property not observed in SCAPs mainly at late passage. However, since clonogenicity was shown to be improved at low [O_2_], we performed the test at 21% O_2_ to determine whether cells maintained their clonogenicity advantage. We observed that clonogenicity of SCAPs was variable among the three tested individuals and that the switch from 3% to 21% O_2_ was deleterious at late passage for SCAP clonogenicity. A direct comparison of clonogenicity efficiency of SCAPs grown at 3% or 21% O_2_ should help to assess whether low [O_2_] maintains better the self-renewal capacity of SCAPs. SSEA4, which is highly expressed in human ESCs and iPSCs, is a stemness marker enriched in cells maintaining their stemness properties [60,61]. It might be relevant to determine whether SCAPs sorted with positive selection for both markers maintain their stemness properties better. Further studies are required to better understand if there is a link between high CD49f and SSEA4 expression and proliferation in SCAPs.

### 4.2. Mesodermal Differentiation Potential of SCAPs is Maintained at Early but Not Late Passages under Low [O_2_]

Differentiation properties of SCAPs towards mesodermal lineages were equivalent at both [O_2_] at early passages (up to P7) but was restricted to chondrogenesis at late passages under 3% O_2_, in agreement with previous reports showing that osteogenesis was reduced under low [O_2_] [15,62]. The media used for these experiments have been optimized under 21% O_2_ and might lack important components essential for efficient differentiation at late passage. However, at 3% O_2_, SCAPs still proliferated efficiently, and did not reach the plateau of their growth even after 28 passages. However, they became unresponsive to various compounds in the osteo- and adipo- differentiation media. The composition of SCAP secretome could change after many passages in vitro and further analyses will better characterize the differentiation restriction of aging SCAP cells [63]. In addition, it might be relevant to determine the expression profile of a new long non-coding RNA (lncRNA STL) in early and late UBx-SCAP according to the recent report showing its involvement in osteogenic differentiation in the DPSC model [15]. In addition, highly proliferative SCAPs grown at 3% O_2_ should now be challenged for their potential to differentiate to neuronal or endodermal lineages.

### 4.3. Active Autophagy Remains for Long Periods in SCAPs

Autophagy is known as a stress-induced mechanism that allows cells to recycle macromolecules and organelles to cope with stress conditions and thereby promote cell survival [26]. Hypoxia is one of the classical inducers of autophagy in cell culture [64,65]. Therefore, it was relevant to investigate autophagy activity in cells which have never been in contact with ambient air and which have only been exposed to low [O_2_]. As expected, we found that the activation of autophagy was associated with high LC3 puncta and with the induction of BNIP3 (a key mitophagy player) in SCAPs switched from 21% to 3% O_2_ (Figure 6). Interestingly, high expression of BNIP3 and active autophagic flux was also observed in SCAPs isolated and expanded at 3% O_2_ at both early and late passages. An active autophagic flux, but no BNIP3 expression, was also observed in SCAPs isolated and expanded at 21% O_2_ strongly indicating that autophagy could fundamentally regulate SCAP physiology at both [O_2_]. A recent report emphasizes the impact of positive autophagy in a rat spinal cord injury model challenged with neural stem cell derived vesicles previously induced to undergo autophagy. Autophagy also positively regulates the therapeutic potential of MSCs for spinal cord injury [66,67]. In SCAPs, BNIP3 expression is neither associated with apoptosis nor with proliferation defects, in contrast to data presented in previous reports [68,69]. Therefore, high autophagic flux associated with a potential mitophagy induction under low [O_2_], could be protective to SCAPs, which remain healthy without any sign of apoptosis after at least one month of culture under low [O_2_] conditions. Interestingly, we have also observed, by double staining of cells with LC3 and BNIP3, heterogeneity in the expression of these markers. Indeed, all situations have been observed with cells expressing only LC3 or BNIP3 or both proteins, but not in the same structure, or neither (observed in few cells). This observation questions the potential involvement of BNIP3 in mitophagy since LC3 and BNIP3 usually colocalize when an active mitophagy process is engaged. Further investigation is needed to determine the significance of the heterogeneous expression of autophagy/mitophagy-involved markers in SCAP physiology. Nevertheless, our work suggests that autophagy is not an acute stress response in SCAPs and its impact in stemness and proliferation of cells should be further analyzed.

## 5. Conclusions

In this study, we have produced SCAP banks from EXP I, II and III and characterized many features of the cells which are now available for further fundamental and applied research. SCAPs directly isolated at 3% O_2_ did not reach their exhaustion phase, up to 24 passages, in contrast with SCAPs isolated and grown at 21% O_2_ that we could not keep growing after 20 passages. However, at low [O_2_] and late passages, SCAPs lost some of their stemness properties in classical differentiation medium, that was established for experiments performed at 21% O_2_. Future work with a larger sampling of cells will aim at deciphering the role of autophagy in increased proliferation of SCAPs grown at 3% O_2_. A key future challenge will be also to determine how pre-conditioned SCAPs at 3% O_2_ survive after transplantation in experimental animal models and to understand better the mechanism of their adaptation to [O_2_] in vivo.

## Figures and Tables

**Table 1:** List of all antibodies used in this study.

Antibody	Antigen Targeted	Supplier	Host Isotype	Clone Reference	Concentration (Stock Sol.)
Antibodies for Flow Cytometry Analyses					
Isotype PE	None	eBioscience	Mouse IgG1, κ	17-4714-42	0.1 µg/µL
Isotype APC	None	eBioscience	Mouse IgG1, κ	17-4714-42	0.1 µg/µL
CD24 APC	CD24 GPI-anchored glycoprotein	eBioscience	Mouse IgG1, κ	eBioSN3 17-0247-42	0.05 µg/µL
CD49f PE	CD49f Integrin α6	eBioscience	Rat IgG2a, κ	eBioGoH3 12-0495-81	0.2 µg/µL
CD73 PE	CD73 ecto-5′-nucléotidase	eBioscience	Mouse IgG1, κ	AD2 12-0739-42	0.025 µg/µL
CD90 PE	CD90 Thy1	eBioscience	Mouse IgG1, κ	eBio5E10 12-0909-42	0.05 µg/µL
CD105 APC	CD105 endoglin	eBioscience	Mouse IgG1, κ	SN6 17-1057-42	0.05 µg/µL
PDGFβ PE	PDGFRβ Platelet Derived Growth Factorβ	Biolegend	Mouse IgG1, κ	18A2 323605	400 µg/mL
CD271 PE	CD271 nerve growth factor receptor	eBioscience	Mouse IgG1, κ	ME20.4 12-9400-42	0.05 µg/µL
SSEA4 APC	SSEA-4, stage specific embryonic antigen 4	BioLegend	Mouse IgG3, κ	MC-813-70 330417	50 µg/mL
CD45 APC	LCA, leucocyte common antigen	eBioscience	Mouse IgG1, κ	HI30-17-0459-42	0.012 µg/µL
CD34 APC	Mucosialin, CD34 antigen	eBioscience	Mouse IgG1, κ	4H11-17-0349-42	0.05 µg/µL
Antibodies for Immunolabelling Studies					
LC3	Microtubule-associated protein light chain 3	MBL	Mouse hybridoma IgG1, κ	M152-3	2 µg/µL
BNIP3	Bcl2/adenovirus 19-kDa-interacting protein 3	Abcam	Rabbit monoclonal	Ab109362	1.2 µg/µL
Alexa 488 Goat	Mouse IgG (H + L)	Invitrogen	Goat Polyclonal	A11001	2 µg/µL
Alexa 568 Goat	Rabbit IgG (H + L)	Invitrogen	Goat Polyclonal	A11036	2 µg/µL
Antibodies for Western Blot					
Nanog	Nanog	Abcam	Rabbit Monoclonal	EPR2027	0.3 mg/mL
Oct4	Oct4 Transcription factor that binds to an octamer motif	Abcam	IgG Rabbit Polyclonal	Ab19857	1 mg/mL
ERK2	Extracellular Regulated Kinase 2	Santa Cruz Biotechnol.	IgG Rabbit Polyclonal	Sc154	0.2 mg/mL
Ac II-HRP	Rabbit IgG (H+L)	Jackson Immuno Research	Goat Peroxidase Conjugated	111-035-144	0.8 mg/mL

## Supplementary Materials

The following are available online at https://www.mdpi.com/2073-4409/8/12/1485/s1, Figure S1: Cumulative population doubling of one patient of the Experience II, Figure S2: Analysis of cell cycle phases, 4 days after seeding UBx-SCAP-1 (from EXP III) and SCAPs from EXP II, as indicated, Figure S3: Characterization of the autophagy process: double staining with LC3 and BNIP3 showing heterogeneous expression of these markers in UBx-SCAP.

## References

[1] Kobolak J., Dinnyes A., Memic A., Khademhosseini A., Mobasheri A. (2016). Mesenchymal stem cells: Identification, phenotypic characterization, biological properties and potential for regenerative medicine through biomaterial micro-engineering of their niche. Methods San Diego Calif..

[2] Liu J., Yu F., Sun Y., Jiang B., Zhang W., Yang J., Xu G.-T., Liang A., Liu S. (2015). Concise reviews: Characteristics and potential applications of human dental tissue-derived mesenchymal stem cells. Stem Cells Dayt. Ohio.

[3] Rastegar F., Shenaq D., Huang J., Zhang W., Zhang B.-Q., He B.-C., Chen L., Zuo G.-W., Luo Q., Shi Q. (2010). Mesenchymal stem cells: Molecular characteristics and clinical applications. World J. Stem Cells.

[4] Hoogduijn M.J., Lombardo E. (2019). Concise Review: Mesenchymal Stromal Cells Anno 2019: Dawn of the Therapeutic Era?. Stem Cells Transl. Med..

[5] Huang G.T.-J., Sonoyama W., Liu Y., Liu H., Wang S., Shi S. (2008). The hidden treasure in apical papilla: The potential role in pulp/dentin regeneration and bioroot engineering. J. Endod..

[6] Sonoyama W., Liu Y., Yamaza T., Tuan R.S., Wang S., Shi S., Huang G.T.-J. (2008). Characterization of the apical papilla and its residing stem cells from human immature permanent teeth: A pilot study. J. Endod..

[7] Bakopoulou A., About I. (2016). Stem Cells of Dental Origin: Current Research Trends and Key Milestones towards Clinical Application. Stem Cells Int..

[8] Kang J., Fan W., Deng Q., He H., Huang F. (2019). Stem Cells from the Apical Papilla: A Promising Source for Stem Cell-Based Therapy. BioMed. Res. Int..

[9] Nada O.A., El Backly R.M. (2018). Stem Cells from the Apical Papilla (SCAP) as a Tool for Endogenous Tissue Regeneration. Front. Bioeng. Biotechnol..

[10] Leyendecker Junior A., Gomes Pinheiro C.C., Lazzaretti Fernandes T., Franco Bueno D. (2018). The use of human dental pulp stem cells for in vivo bone tissue engineering: A systematic review. J. Tissue Eng..

[11] Grayson W.L., Zhao F., Bunnell B., Ma T. (2007). Hypoxia enhances proliferation and tissue formation of human mesenchymal stem cells. Biochem. Biophys. Res. Commun..

[12] Ivanovic Z. (2009). Hypoxia or in situ normoxia: The stem cell paradigm. J. Cell. Physiol..

[13] Ivanovic Z., Vlaski-Lafarge M. (2016). Harnessing anaerobic nature of stem cells for use in regenerative medicine. Anaerobiosis and Stemness: An Evolutionary Paradigm.

[14] Gorin C., Rochefort G.Y., Bascetin R., Ying H., Lesieur J., Sadoine J., Beckouche N., Berndt S., Novais A., Lesage M. (2016). Priming Dental Pulp Stem Cells with Fibroblast Growth Factor-2 Increases Angiogenesis of Implanted Tissue-Engineered Constructs Through Hepatocyte Growth Factor and Vascular Endothelial Growth Factor Secretion. Stem Cells Transl. Med..

[15] Shi R., Yang H., Lin X., Cao Y., Zhang C., Fan Z., Hou B. (2019). Analysis of the characteristics and expression profiles of coding and noncoding RNAs of human dental pulp stem cells in hypoxic conditions. Stem Cell Res. Ther..

[16] D’Ippolito G., Diabira S., Howard G.A., Menei P., Roos B.A., Schiller P.C. (2004). Marrow-isolated adult multilineage inducible (MIAMI) cells, a unique population of postnatal young and old human cells with extensive expansion and differentiation potential. J. Cell Sci..

[17] D’Ippolito G., Diabira S., Howard G.A., Roos B.A., Schiller P.C. (2006). Low oxygen tension inhibits osteogenic differentiation and enhances stemness of human MIAMI cells. Bone.

[18] Rios C., D’Ippolito G., Curtis K.M., Delcroix G.J.-R., Gomez L.A., El Hokayem J., Rieger M., Parrondo R., de Las Pozas A., Perez-Stable C. (2016). Low Oxygen Modulates Multiple Signaling Pathways, Increasing Self-Renewal, While Decreasing Differentiation, Senescence, and Apoptosis in Stromal MIAMI Cells. Stem Cells Dev..

[19] Lennon D.P., Edmison J.M., Caplan A.I. (2001). Cultivation of rat marrow-derived mesenchymal stem cells in reduced oxygen tension: Effects on in vitro and in vivo osteochondrogenesis. J. Cell. Physiol..

[20] Ren H., Cao Y., Zhao Q., Li J., Zhou C., Liao L., Jia M., Zhao Q., Cai H., Han Z.C. (2006). Proliferation and differentiation of bone marrow stromal cells under hypoxic conditions. Biochem. Biophys. Res. Commun..

[21] Ivanovic Z., Vlaski-Lafarge M. (2016). In situ normoxia versus “Hypoxia”. Anaerobiosis and Stemness: An Evolutionary Paradigm.

[22] Schäfer R., Spohn G., Baer P.C. (2016). Mesenchymal Stem/Stromal Cells in Regenerative Medicine: Can Preconditioning Strategies Improve Therapeutic Efficacy?. Transfus. Med. Hemother..

[23] Choi J.R., Pingguan-Murphy B., Wan Abas W.A.B., Noor Azmi M.A., Omar S.Z., Chua K.H., Wan Safwani W.K.Z. (2014). Impact of low oxygen tension on stemness, proliferation and differentiation potential of human adipose-derived stem cells. Biochem. Biophys. Res. Commun..

[24] Fotia C., Massa A., Boriani F., Baldini N., Granchi D. (2015). Prolonged exposure to hypoxic milieu improves the osteogenic potential of adipose derived stem cells. J. Cell. Biochem..

[25] Mas-Bargues C., Sanz-Ros J., Román-Domínguez A., Inglés M., Gimeno-Mallench L., El Alami M., Viña-Almunia J., Gambini J., Viña J., Borrás C. (2019). Relevance of Oxygen Concentration in Stem Cell Culture for Regenerative Medicine. Int. J. Mol. Sci..

[26] Boya P., Codogno P., Rodriguez-Muela N. (2018). Autophagy in stem cells: Repair, remodelling and metabolic reprogramming. Dev. Camb. Engl..

[27] Meijer A.J., Codogno P. (2004). Regulation and role of autophagy in mammalian cells. Int. J. Biochem. Cell Biol..

[28] Sotthibundhu A., Promjuntuek W., Liu M., Shen S., Noisa P. (2018). Roles of autophagy in controlling stem cell identity: A perspective of self-renewal and differentiation. Cell Tissue Res..

[29] Guan J.-L., Simon A.K., Prescott M., Menendez J.A., Liu F., Wang F., Wang C., Wolvetang E., Vazquez-Martin A., Zhang J. (2013). Autophagy in stem cells. Autophagy.

[30] Tanida I., Ueno T., Kominami E. (2004). LC3 conjugation system in mammalian autophagy. Int. J. Biochem. Cell Biol..

[31] Semenza G.L. (2008). Mitochondrial autophagy: Life and breath of the cell. Autophagy.

[32] Ney P.A. (2015). Mitochondrial autophagy: Origins, significance, and role of BNIP3 and NIX. Biochim. Biophys. Acta.

[33] Lee H.J., Jung Y.H., Choi G.E., Ko S.H., Lee S.-J., Lee S.H., Han H.J. (2017). BNIP3 induction by hypoxia stimulates FASN-dependent free fatty acid production enhancing therapeutic potential of umbilical cord blood-derived human mesenchymal stem cells. Redox Biol..

[34] Martin D.E., De Almeida J.F.A., Henry M.A., Khaing Z.Z., Schmidt C.E., Teixeira F.B., Diogenes A. (2014). Concentration-dependent effect of sodium hypochlorite on stem cells of apical papilla survival and differentiation. J. Endod..

[35] Devillard R., Rémy M., Kalisky J., Bourget J.-M., Kérourédan O., Siadous R., Bareille R., Amédée-Vilamitjana J., Chassande O., Fricain J.-C. (2017). In vitro assessment of a collagen/alginate composite scaffold for regenerative endodontics. Int. Endod. J..

[36] Campbell J.H., Campbell G.R. (1993). Culture techniques and their applications to studies of vascular smooth muscle. Clin. Sci..

[37] Greenwood S.K., Hill R.B., Sun J.T., Armstrong M.J., Johnson T.E., Gara J.P., Galloway S.M. (2004). Population doubling: A simple and more accurate estimation of cell growth suppression in the in vitro assay for chromosomal aberrations that reduces irrelevant positive results. Environ. Mol. Mutagen..

[38] Duval D., Trouillas M., Thibault C., Dembele D., Diemunsch F., Reinhardt B., Mertz A.L., Dierich A., Boeuf H. (2006). Apoptosis and differentiation commitment: Novel insights revealed by gene profiling studies in mouse embryonic stem cells. Cell Death Differ..

[39] Hammoud A.A., Kirstein N., Mournetas V., Darracq A., Broc S., Blanchard C., Zeineddine D., Mortada M., Boeuf H. (2016). Murine Embryonic Stem Cell Plasticity Is Regulated through Klf5 and Maintained by Metalloproteinase MMP1 and Hypoxia. PLoS ONE.

[40] Smyrek I., Stelzer E.H.K. (2017). Quantitative three-dimensional evaluation of immunofluorescence staining for large whole mount spheroids with light sheet microscopy. Biomed. Opt. Express.

[41] Coronado D., Godet M., Bourillot P.-Y., Tapponnier Y., Bernat A., Petit M., Afanassieff M., Markossian S., Malashicheva A., Iacone R. (2013). A short G1 phase is an intrinsic determinant of naïve embryonic stem cell pluripotency. Stem Cell Res..

[42] Elabd C., Ichim T.E., Miller K., Anneling A., Grinstein V., Vargas V., Silva F.J. (2018). Comparing atmospheric and hypoxic cultured mesenchymal stem cell transcriptome: Implication for stem cell therapies targeting intervertebral discs. J. Transl. Med..

[43] Antebi B., Rodriguez L.A., Walker K.P., Asher A.M., Kamucheka R.M., Alvarado L., Mohammadipoor A., Cancio L.C. (2018). Short-term physiological hypoxia potentiates the therapeutic function of mesenchymal stem cells. Stem Cell Res. Ther..

[44] Aguilar P., Lertchirakarn V. (2016). Comparison of stem cell behaviors between indigenous high and low-CD24 percentage expressing cells of stem cells from apical papilla (SCAPs). Tissue Cell.

[45] Wang S., Mo M., Wang J., Sadia S., Shi B., Fu X., Yu L., Tredget E.E., Wu Y. (2018). Platelet-derived growth factor receptor beta identifies mesenchymal stem cells with enhanced engraftment to tissue injury and pro-angiogenic property. Cell. Mol. Life Sci..

[46] Henderson J.K., Draper J.S., Baillie H.S., Fishel S., Thomson J.A., Moore H., Andrews P.W. (2002). Preimplantation human embryos and embryonic stem cells show comparable expression of stage-specific embryonic antigens. Stem Cells.

[47] Yu K.-R., Yang S.-R., Jung J.-W., Kim H., Ko K., Han D.W., Park S.-B., Choi S.W., Kang S.-K., Schöler H. (2012). CD49f enhances multipotency and maintains stemness through the direct regulation of OCT4 and SOX2. Stem Cells Dayt. Ohio.

[48] Alvarez R., Lee H.-L., Hong C., Wang C.-Y. (2015). Single CD271 marker isolates mesenchymal stem cells from human dental pulp. Int. J. Oral Sci..

[49] Takahashi K., Tanabe K., Ohnuki M., Narita M., Ichisaka T., Tomoda K., Yamanaka S. (2007). Induction of pluripotent stem cells from adult human fibroblasts by defined factors. Cell.

[50] Sbrana F.V., Cortini M., Avnet S., Perut F., Columbaro M., De Milito A., Baldini N. (2016). The Role of Autophagy in the Maintenance of Stemness and Differentiation of Mesenchymal Stem Cells. Stem Cell Rev..

[51] Cossu G., Birchall M., Brown T., De Coppi P., Culme-Seymour E., Gibbon S., Hitchcock J., Mason C., Montgomery J., Morris S. (2018). Lancet Commission: Stem cells and regenerative medicine. Lancet.

[52] Cipolleschi M.G., Rovida E., Ivanovic Z., Praloran V., Olivotto M., Sbarba P.D. (2000). The expansion of murine bone marrow cells preincubated in hypoxia as an in vitro indicator of their marrow-repopulating ability. Leukemia.

[53] Hammoud M., Vlaski M., Duchez P., Chevaleyre J., Lafarge X., Boiron J.-M., Praloran V., Brunet De La Grange P., Ivanovic Z. (2012). Combination of low O_2_ concentration and mesenchymal stromal cells during culture of cord blood CD34(+) cells improves the maintenance and proliferative capacity of hematopoietic stem cells. J. Cell. Physiol..

[54] Leroux L., Descamps B., Tojais N.F., Séguy B., Oses P., Moreau C., Daret D., Ivanovic Z., Boiron J.-M., Lamazière J.-M.D. (2010). Hypoxia preconditioned mesenchymal stem cells improve vascular and skeletal muscle fiber regeneration after ischemia through a Wnt4-dependent pathway. Mol. Ther. J. Am. Soc. Gene Ther..

[55] Grau-Monge C., Delcroix G.J.-R., Bonnin-Marquez A., Valdes M., Awadallah E.L.M., Quevedo D.F., Armour M.R., Montero R.B., Schiller P.C., Andreopoulos F.M. (2017). Marrow-isolated adult multilineage inducible cells embedded within a biologically-inspired construct promote recovery in a mouse model of peripheral vascular disease. Biomed. Mater..

[56] Niculescu C., Ganguli-Indra G., Pfister V., Dupé V., Messaddeq N., De Arcangelis A., Georges-Labouesse E. (2011). Conditional ablation of integrin alpha-6 in mouse epidermis leads to skin fragility and inflammation. Eur. J. Cell Biol..

[57] De Arcangelis A., Hamade H., Alpy F., Normand S., Bruyère E., Lefebvre O., Méchine-Neuville A., Siebert S., Pfister V., Lepage P. (2017). Hemidesmosome integrity protects the colon against colitis and colorectal cancer. Gut.

[58] Qiryaqoz Z., Timilsina S., Czarnowski D., Krebsbach P.H., Villa-Diaz L.G. (2019). Identification of biomarkers indicative of functional skeletal stem cells. Orthod. Craniofac. Res..

[59] Yang Z., Dong P., Fu X., Li Q., Ma S., Wu D., Kang N., Liu X., Yan L., Xiao R. (2015). CD49f Acts as an Inflammation Sensor to Regulate Differentiation, Adhesion, and Migration of Human Mesenchymal Stem Cells. Stem Cells Dayt. Ohio.

[60] Inoue H., Nagata N., Kurokawa H., Yamanaka S. (2014). iPS cells: A game changer for future medicine. EMBO J..

[61] Takahashi K., Okita K., Nakagawa M., Yamanaka S. (2007). Induction of pluripotent stem cells from fibroblast cultures. Nat. Protoc..

[62] Pattappa G., Thorpe S.D., Jegard N.C., Heywood H.K., de Bruijn J.D., Lee D.A. (2013). Continuous and uninterrupted oxygen tension influences the colony formation and oxidative metabolism of human mesenchymal stem cells. Tissue Eng. Part. C Methods.

[63] Nawaz M., Fatima F., Vallabhaneni K.C., Penfornis P., Valadi H., Ekström K., Kholia S., Whitt J.D., Fernandes J.D., Pochampally R. (2016). Extracellular Vesicles: Evolving Factors in Stem Cell Biology. Stem Cells Int..

[64] Semenza G.L. (1999). Regulation of mammalian O2 homeostasis by hypoxia-inducible factor 1. Annu. Rev. Cell Dev. Biol..

[65] Semenza G.L. (2014). Oxygen sensing, hypoxia-inducible factors, and disease pathophysiology. Annu. Rev. Pathol..

[66] Chen X., He Y., Lu F. (2018). Autophagy in Stem Cell Biology: A Perspective on Stem Cell Self-Renewal and Differentiation. Stem Cells Int..

[67] Ma F., Li R., Tang H., Zhu T., Xu F., Zhu J. (2019). Regulation of autophagy in mesenchymal stem cells modulates therapeutic effects on spinal cord injury. Brain Res..

[68] Esteban-Martínez L., Boya P. (2017). BNIP3L/NIX-dependent mitophagy regulates cell differentiation via metabolic reprogramming. Autophagy.

[69] Singh A., Azad M., Shymko M.D., Henson E.S., Katyal S., Eisenstat D.D., Gibson S.B. (2018). The BH3 only Bcl-2 family member BNIP3 regulates cellular proliferation. PLoS ONE.

