# Erratum: Rémy et al. Isolation and Culture of Human Stem Cells from Apical Papilla under Low Oxygen Concentration Highlight Original Properties. *Cells* 2019, *8*, 1485

**DOI:** 10.3390/cells10050988

**Published:** 2021-04-23

**Authors:** Murielle Rémy, Francesca Ferraro, Pierre Le Salver, Sylvie Rey, Elisabeth Genot, Mojgan Djavaheri-Mergny, Noélie Thébaud, Claudine Boiziau, Hélène Boeuf

**Affiliations:** 1 INSERM, University Bordeaux, U1026, Laboratory for the Bioengineering of Tissues, F-33000 Bordeaux, France; murielle.remy@inserm.fr (M.R.); francesca.ferraro.bio@gmail.com (F.F.); pierre.le.salver@gmail.com (P.L.S.); sylvie.rey@inserm.fr (S.R.); noelie.thebaud@inserm.fr (N.T.); claudine.boiziau@inserm.fr (C.B.); 2 INSERM, University Bordeaux, U1045, F-33000 Bordeaux, France; elisabeth.genot@u-bordeaux.fr; 3 INSERM, University Bordeaux, U1218 Action, F-33000 Bordeaux, France; mojgan.mergny@inserm.fr

The authors wish to make the following change to their paper [1].

Figure 2A should be replaced with the correct annotations for each curve. The correct Figure 2 is shown below.

And in Table 1, the reference of the isotype PE is as follows: 12-4714-42.

We apologize for this error and state that the scientific conclusions are unaffected. The original article has been updated.

## Figures and Tables

**Figure 2:**
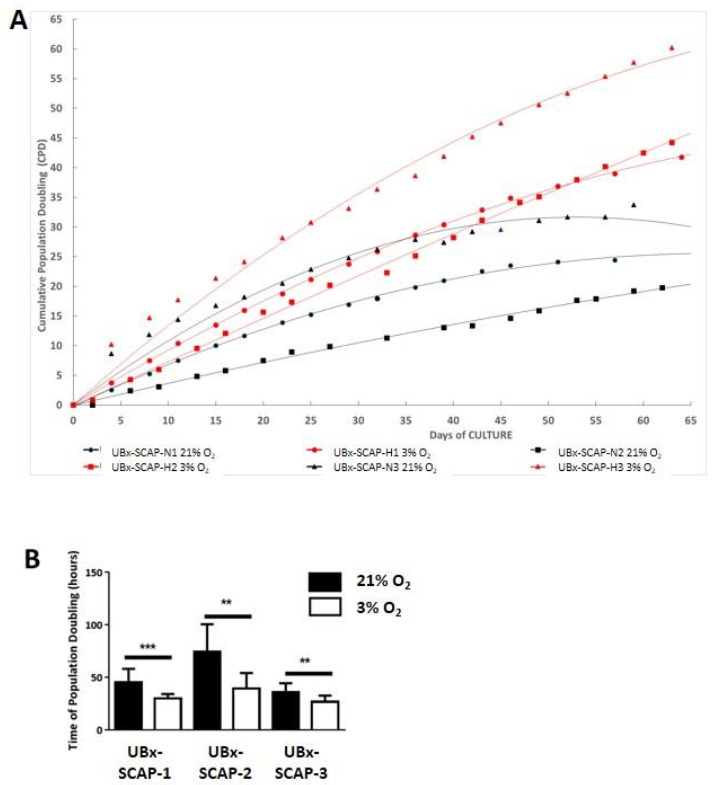
Proliferative advantage of UBx-SCAP isolated under 3% O_2_ in comparison with ambient air (21% O_2_). (**A**) At each passage of SCAPs from EXP III, 0.4 (under 3% O_2_) or 0.8 (under 21% O_2_) millions of cells were seeded in a 75 cm^2^ flask and counted after three or four days. Cumulative population doublings (CPD) were plotted for each individual refered to UBx-SCAP-N1, N2 and N3 (21% O_2_) and UBx-SCAP-H1, H2 and H3 (3% O_2_), up to 65 days. (**B**) The mean of time of population doubling for the first 10 passages, for each individual at 21% and 3% O_2_ is plotted with standard deviation. Statistical analyses were done with a Mann-Whitney test. ** *p* < 0.01. *** *p* < 0.001.
