# Supplementary material for: Isolation and Culture of Human Stem Cells from Apical Papilla under Low Oxygen Concentration Highlight Original Properties
Source: Cells. 2019 Nov 21;8(12):1485. doi: 10.3390/cells8121485 (PMC6952825; doi:10.3390/cells8121485)
Supplement: Supplementary file 1 [file cells-08-01485-s001.docx]

**SUPPLEMENTARY FIGURES**


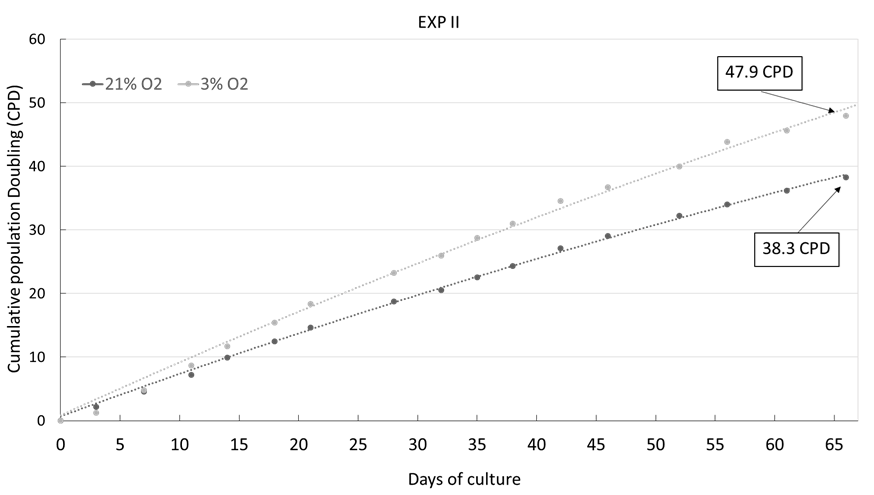


**Figure S1:** Cumulative population doubling of one patient of the Experience II.


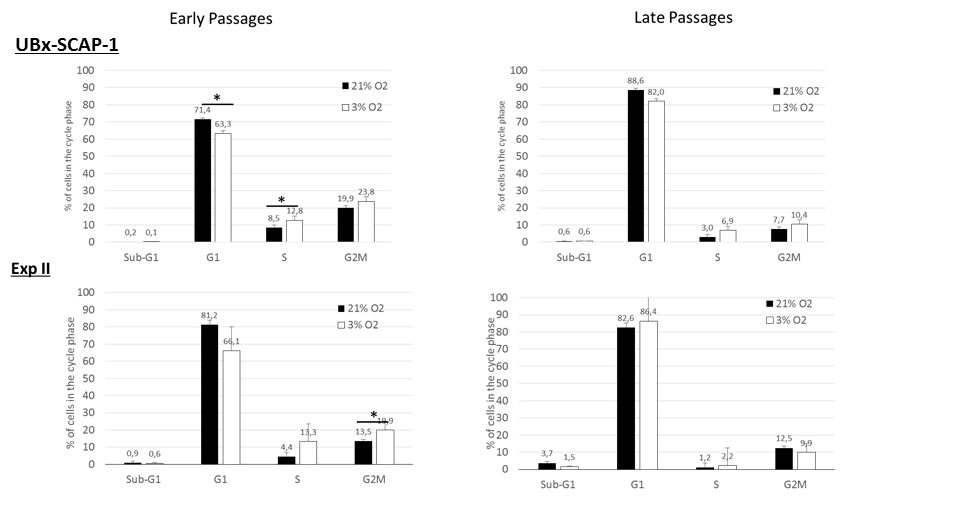


**Figure S2:** Analysis of cell cycle phases, 4 days after seeding UBx-SCAP-1 (from EXP III) and SCAPs from EXP II, as indicated.

**
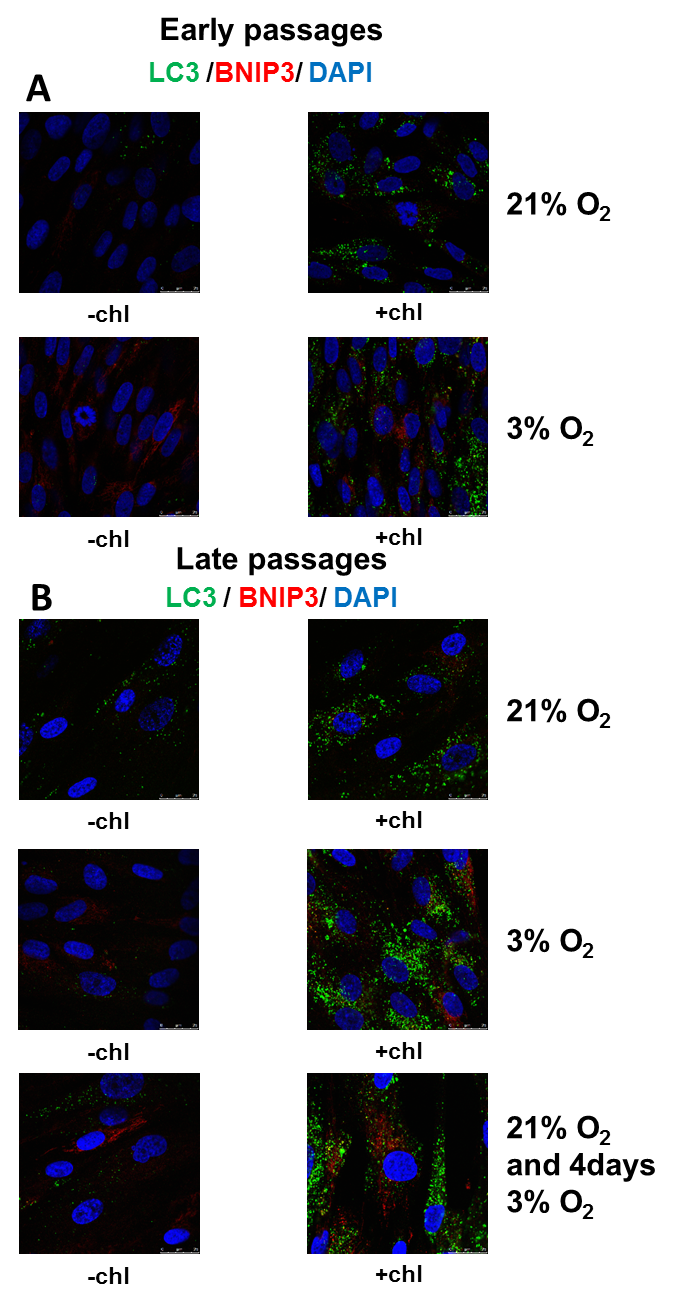
**

**Figure S3: Characterization of the autophagy process: double staining with LC3 and BNIP3 showing heterogeneous expression of these markers in UBx-SCAP.**

Early and late passages of UBx-SCAPs, derived and amplified either at 21% O_2_ or at 3% O_2_, or derived and amplified at 21% and switched for 4 days at 3%O_2_, were labelled with LC3 and Bnip3 antibodies as indicated. For each individual and condition, before labelling, cells were treated (+chl) or not (-chl) for 5h with 20 µM Chloroquine. Representative pictures of double staining of UBx-SCAP-3 are shown here. The same pictures, separated for each antibody with the Image J software, are presented in Figure 6. Scale bar is 25 µm.
